# The LH/hCG Axis in Endometrial Cancer: A New Target in the Treatment of Recurrent or Metastatic Disease

**DOI:** 10.1155/2010/486164

**Published:** 2010-07-15

**Authors:** A. Arcangeli, I. Noci, A. Fortunato, G. F. Scarselli

**Affiliations:** ^1^Department of Experimental Pathology and Oncology, Unversity of Florence, Viale GB Morgagni, 50, 50134 Firenze, Italy; ^2^Department of Gynaecology, Perinatology and Human Reproduction, University of Florence, Viale GB Morgagni, 85, 50134 Firenze, Italy

## Abstract

Endometrial cancer (EC) is a hormone-dependent cancer that currently represents the most frequent malignancy of the female reproductive tract. The involvement of steroid hormones in EC etiology and progression has been reported. More recently, gonadotropins, and, in particular LH/hCG, are emerging as novel regulators of tumor progression. In the present review, we discuss the role of the LH/hCG axis (i.e. LH/hCG and its receptors, LH/hCG-R) in both gonadal and nongonadal tissues, in physiological and neoplastic conditions. In cancer cells, LH/hCG mainly controls cell proliferation and apoptosis. In particular, in EC LH/hCG improves cell invasiveness, through a mechanism which involves the LH/hCG-R, which in turn activate protein kinase A and modulate integrin adhesion receptors. Indeed, the LH/hCG-R mRNA is expressed in primary ECs and this expression correlates with LH/hCG-induced cell invasiveness in vitro. These results lead to hypothesize that recurrent and metastatic ECs, which express LH/hCG-R, could benefit from therapies aimed at decreasing LH levels, through Gn-RH analogues. Hence, the LH/hCG axis could represent a prognostic factor and a new therapeutic target in EC.

## 1. Introduction

Endometrial cancer (EC) is currently the most frequent malignancy of the female reproductive tract [1] with a tendency to increase its incidence during the last decade [2, 3]. Approximately 75% of EC cases are diagnosed with the tumor confined to the uterine corpus [2, 3], but after primary surgery 15% to 20% of these tumors recur and have limited response to systemic therapy. The most common basis for determining risk of recurrent disease has been the categorization of EC into 2 subtypes: type I EC is associated with good prognosis, low stage and grade, and endometrioid histology; type II EC is, in contrast, characterized by high stage and grade, nonendometrioid histology and poor prognosis. The prognostic value of this distinction is limited, however, because up to 20% of type I cancer recur, and half of type II cancers do not [3]. Moreover, the molecular basis of the distinction between type I and II cancer is understood only partially. Type I cancer is associated with hyperestrogenic risk factors, is more often estrogen and progesterone receptor positive, microsatellite unstable, and displays mutations in KRAS or PTEN. Conversely, type II cancer is more often aneuploid and harbors alterations in CDKN2A, TP53, and ERBB2 [4]. Such molecular alterations, despite of prognostic value, have not provided a basis forimproved therapy [5]. 

Hormone (estrogen and progesterone) receptor status influences the choice of treatment especially in metastatic disease. Indeed estrogen secretion, especially when occurring without progesterone secretion, is apparently one of the most relevant etiologic factors in EC [6]. In fact, unopposed and prolonged exposure to estrogens can exert potent mitogenic effects on the endometrial surface epithelium, thus contributing to the malignant transformation of the latter [7]. However, as stated above, most aggressive tumors are often receptor negative [2]. In particular, those cancers arising in the postmenopause, which are often more aggressive, are also apparently unlinked to estrogen secretion [8]. It was hypothesized that the latter types of EC might be sensitive to the elevated levels of luteinizing hormone/human chorionic gonadotropin (LH/hCG) that characterize the post-menopause. 

The present review aims at updating the reader about some current trends in the field. In particular, we will focus on a brief overview of the single elements of the LH/hCG axis and its relevance in nongonadal tissues in physiological conditions. Moreover, we will briefly review the role of the LH/hCG axis in human cancers, with particular emphasis to EC. Finally, we will provide new perspectives for treatment of EC based on the targeting of the LH/hCG axis.

## 2. The LH /hCG Axis: LH/hCG, LH/hCG-R, and Intracellular Signalling

LH and hCG are structurally related glycoprotein hormones (GPH) produced by the pituitary gland and placenta, respectively. Both LH and hCG are heterodimers of noncovalently bound *α* and *β* subunits; hCG has a higher molecular mass and is the most heavily glycosylated among the GPH, resulting in a longer circulating half-life [9]. LH, secreted by anterior pituitary gland, is present in all mammalian species. On the other hand, hCG, which is secreted by placental trophoblasts, is present exclusively in primates although its occurrence in other species, as a modified form, has not been completely ruled out [10]. These two hormones bind the same receptors and have similar biological functions, although hCG is more potent because of its higher receptor binding affinity and its longer circulatory half life. LH and hCG are considered the most potent gonadal regulating hormones, even though there are reports demonstrating their effects in nongonadal tissues (see below).

The receptor for LH/hCG is encoded by a gene, located on chromosome 2, position 2p21 [11, 12]. The genomic sequence of the gene consists of 11 exons separated by 10 introns; the transcription of the LH/hCG-R gene can produce 7 different protein-coding mRNAs [12–14]. Recently, three more alternative splice variants have been found in corpus luteum and luteinized granulosa cells [14]. 

The LH/hCG-R is a member of the G-protein coupled receptor (GPCR) family and its functional activity is mediated by G proteins [6, 15]. The amino acids chain of the mature LH/hCG-R protein is composed by 699 residues with a molecular mass of about 78 kDa [12, 13]. The protein consists of a long extracellular N-terminal domain, a region containing seven transmembrane-spanning sequences connected by alternating extra and intracellular loops, and a short C-terminal cytoplasmatic tail [13, 16]. The extracellular domain is characterized by the presence of a structural motif rich in leucine repeats (LRR) which is involved in modulating ligand-receptor(s) binding [16].

 Such binding determines conformational changes in the protein structure that in turn induces the activation of intracellular signalling. In particular, both LH and hCG stimulate adenylate cyclase on the internal membrane, which in turn converts adenosine triphosphate (ATP) into cyclic adenosine monophosphate (cAMP). Cyclic AMP stimulates the activation of an inactive Protein Kinase A (PKA), which, besides other actions, stimulates steroidogenesis in the mitochondria of the target cell by transforming cholesterol into pregnenolone. Other actions of LH/hCG include the induction of proteolytic enzymes, prostaglandin synthesis, inhibin production, induction of 17 beta-hydroxysteroid dehydrogenase, and changes in gene metabolism [17]. LH and hCG can also trigger the PLC/inositide tris-phosphate transduction pathway [18, 19]; the cell type and the amount of the receptor on the plasma membrane can influence the prevalent activation of either the signalling pathways [20]. In porcine endometrial cells, the LH hormone activates both cAMP/PKA and PLC/inositide tris-phosphate pathways [21]. Moreover, LH acting through the Akt and Erk pathways on theca cells plays a relevant function *in vitro *and follicle growth and development [22].

## 3. Expression and Role of the LH/hCG Axis in Extragonadal Tissues

By acting through the transduction mechanisms described above, LH and hCG regulate ovarian steroidogenesis, but have also been shown to exert various effects on nongonadal tissues, such as endometrium, myometrium, and fallopian tubes. 

In the ovary, the main function of GTs is well known. According to the “two cells-two gonadotropins” theory, LH is acting on theca cells during ovarian follicle growth. At first, the synthesis of androgens is induced, then androgens are transformed in estrogens in the compartment of granulosa cells through an enzymatic reaction which is dependent on FSH [23]. Besides ovary, receptors for both LH and FSH were identified in different organs of the female reproductive apparatus, and in particular in the endometrium [24]. The presence of LH/hCG-Rs was shown for the first time by Reshef et al. [24] in the uterus of nonpregnant women by immunohistochemistry, an observation subsequently confirmed using different techniques [25, 26]. Collectively, LH/hCG-Rs have been identified in epithelial and stromal cells of the endometrium as well in smooth muscle cells of myometrium and uterine vessel. The expression of LH/hGC-R varied during the women's cycle phase, with the maximal expression occurring during the luteal phase, and a main localization in the luminal and glandular epithelial cells of endometrium [27]. The attainment of the highest values of LH/hGC-R correlated, also on a time scale, with the triggering of cyclo-oxygenase-2 (COX-2) and placental growth factor (PlGF) production. This finding suggests the hypothesis that LH could play a pivotal role at the beginning of luteolysis [28]. Moreover, LH could regulate the local metabolism of estrogens and progestins, acting through the cAMP pathway [29].

Specific receptors for LH/hCG have also been identified in the myometrium of several animal species, including humans [24]. In this tissue, LH/hCG apparently acts through the LH/hCG-R-dependent activation of both the c-AMP and phospholipase C transduction pathways [20, 21]. It was proposed that the triggering of adenyl cyclase could determine an activation of COX-2, which in turn should induce an increase of the synthesis of either prostaglandin (PG)E, with an ensuing muscle relaxation, or PGF, which determines the contraction of the uterine musculature [28]. Also in this case, the highest expression of the LH/hCG-R occurred during the luteal phase of the cycle, and paralleled an increased synthesis of PGE2. 

The LH/hCG-R is also expressed in the internal mucosa and smooth muscle vascular cells of fallopian tubes [30]. *In vitro* studies performed on explanted fallopian tissues showed that LH addition produces a dramatic reduction of tube motility [31]. This suggests that LH/hCG-R stimulation could contribute to the quiescent state of tube's muscles after ovulation, in turn favouring both oocyte fertilization and embryo movements along the tube to reach the endometrial cavity.

## 4. Expression and Role of the LH/hCG Axis in EC

Increasing evidence suggests that the action of LH and hCG might also contribute to the malignant transformation of human cells, by promoting either promitogenic or antiapoptotic effects. For example, LH/hCG-R may be involved in the progression of some ovarian and breast cancers [32]. In addition, the expression of LH/hCG-R has been demonstrated in endometriosis and has been shown to be increased in patients with adenomyosis [33–35]. 

Work from Lin et al. [6] showed that the LH/hCG-R mRNA is also expressed in human ECs, and a notion subsequently reinforced by the demonstration that the addition of LH/hCG regulates proliferation in EC cell lines [7, 36]. In particular, two isoforms of the LH/hCG-R, arising from alternative splicing of the corresponding gene are documented in EC samples [37], as well as in neoplastic ovarian tissues [38]. More recently, we confirmed not only that specific LH/hCG-Rs can be detected in human EC, but also that their expression is apparently related to the cancer grading [39]. On the basis of these findings, as well as of the fact that we determined the effects of LH/hCG in tumor progression of EC, by analyzing the effects of such GTs on the invasion potential of both EC cell lines and primary human EC cells. We showed that human recombinant (hr) LH (as well as hCG) induced a significant increase in cell invasiveness through Matrigel-coated porous membranes in the human EC cell line Hec1A, which expresses the LH/hCG-R. This effect turned out to depend on the hrLH binding to its specific receptor and on the following activation of the cAMP/PKA signaling pathway. Moreover, the hrLH-induced increase in Hec1A invasiveness was dependent upon the functional activation of *β*1 integrin receptors and the subsequent induction of MMP-2 secretion. Interestingly, these mechanisms were found to be also operative in primary EC cells transferred *in vitro*, because hrLH addition produced an increase in cells invasiveness only in those primary EC tumors that expressed the LH/hCG-R. Here again, this effect was dependent on PKA activity [40]. Subsequently, we also provided evidence that the LH/hCG-R mRNA is expressed in the great majority of a cohort of primary ECs and that cells obtained from primary ECs can be triggered to invade a Matrigel layer by LH addition. A good correlation was found between the level of LH/hCG-R mRNA expressed by primary EC and that of LH-induced cell invasiveness *in vitro*. The analysis of cell invasion *in vitro* in response to LH/hCG, allowed us to divide the EC samples into two groups, one with a null or very low response (non-responders = NR) and the other with a significant response to LH (responders = R). The two groups had significantly different levels of LH/hCG-R mRNA expression. These results may contribute to reconcile the conflicting results present in the literature, about the clinical effect of LH analogues in the treatment of recurrent or metastatic EC (see below). 

On the whole, these results open the possibility that GTs could directly regulate tumor progression of EC by regulating tumor invasiveness through the binding to specific receptors exposed on the plasma membrane of EC. This finding opens new therapeutic prospective of recurrent EC.

## 5. Therapeutical Perspectives

Some clinical trials were performed with the aim of treating patients affected by EC with Gonadotropin releasing hormone (Gn-RH) analogues, in order to decrease LH levels. Conflicting results emerged from these studies. In fact, Davies et al. [36], Lhommé et al. [41], and Jeyarajah et al. [42] showed evidence of efficacy in long-term treatments, with response rate ranging from 9% to 57%. On the other hand, Covens et al. [43], Markman et al. [44], and Asbury et al. [45] observed insufficient activity. We provided a contribution in this controversy, reporting a case of a patient affected by EC, primarily treated with a Gn-RH analogue. In fact, in this case, surgical treatment was unfeasible, due to the compromised health of the patient [46]. The therapy was carried out for 6 years and no progression of the disease was observed throughout this period.

Our recent data, reported in the previous paragraph, on the role of LH/hCG in EC invasiveness could contribute to reconcile the conflicting results present in the literature, about the clinical effect of LH analogues in the treatment of recurrent or metastatic EC. In fact we showed that only 35% of patients showed a high expression of LH/hCG mRNA and only these patients responded to exogenous recombinant LH addition by increasing the cell invasion through Matrigel [39]. This could in turn imply that only patients highly positive for LH/hCG-R expression could receive benefits from a therapy aimed at decreasing LH levels, through Gn-RH analogues. On the whole, based on available data, we suggest that therapies, employing Gn-RH analogues, could produce benefits in the treatment of recurrent or metastatic EC, especially in those patients where cancer tissue displays high LH/hCG-R levels. We prospect to analyze herein all the IV stage patients (whose five-year survival is less than 10%) for the expression of LH/hCG mRNA and treat only the high expressors with Gn-RH analogues.
